# Renal Function Profiles in Preterm Neonates With Birth Asphyxia Within the First 24 H of Life

**DOI:** 10.3389/fped.2020.583540

**Published:** 2020-10-30

**Authors:** Yu Zhang, Hui-Hui Zeng

**Affiliations:** Department of Neonatal Intensive Care Unit, Beijing Obstetrics and Gynecology Hospital, Capital Medical University, Beijing, China

**Keywords:** preterm, renal function, profiles, first 24 h of life, asphyxia, gestational age

## Abstract

The characteristics of early renal function in preterm neonates of different gestational ages (GAs) with birth asphyxia (BA) remain unclear. Kidneys are sensitive to oxygen deprivation, and renal insufficiency may occur within 24 h of BA. We aimed to elucidate the renal function profiles within the first 24 h after the development of BA among vulnerable preterm neonates of different GAs. The medical records of 128 preterm neonates born to mothers with normal renal function were retrospectively analyzed. Data regarding the serum creatinine (SCr) and urea nitrogen (BUN) levels in venous blood, estimated creatinine clearance (eCCI) within the first hours after birth, and urinary output (UOP) in the first 24 h after birth were compared between the preterm with BA population and GA-matched population without BA (*n* = 64 and *n* = 64, respectively). Significantly higher SCr levels and lower eCCI were observed in mid-late preterm neonates with BA than in preterm neonates without BA (84.05 versus [vs.] 64.20 μmol/L, z = 4.41, *p* < 0.001; 15.02 vs. 21.30 mL/min/1.73 m^2^, z = 3.57, *p* < 0.001, respectively). Very preterm neonates showed a higher UOP (2.01 vs. 1.66 mL/kg/h, z = 2.01, *p* = 0.045) after the development of BA than before. In preterm neonates with BA, the incidence of SCr > 133 μmol/L, CCI < 16 mL/min/1.73 m^2^ and UOP < 1.0 ml/kg/h, was 10.94%, 62.50%, and 20.31%, respectively. Within 24 h after birth, BA was associated with eCCI < 16 mL/min/1.73 m^2^ (*p* = 0.016, odds ratio = 2.83, 95% confidence interval: 1.210–6.613) in preterm neonates. Different renal function profiles were observed in preterm neonates of different GAs within the first 24 h of life after the development of BA. Candidate therapies based on different renal function statuses will bring these vulnerable patient populations of different GAs closer to receiving precision medicine.

## Introduction

Renal function in neonate changes markedly daily, and different degrees of renal immaturity exist in preterm neonates of different gestational ages (GAs) (1); Thus, evaluating the renal function of the preterm population is challenging. The estimated global preterm birth rate for 2018 ranged between 5 and 18% across 184 countries worldwide (2). Preterm neonates have multiple organ immaturity and high mortality, which increases with decreasing GA (3). Birth asphyxia (BA), a critical condition of transient anoxia experienced by neonates at or around the time of delivery, occurs at 1–10% per 1,000 live births and is more likely to occur in preterm neonates (4).

BA leads to dysfunction of the central nervous system and multiple organs, including the heart, lungs, kidneys, and bowel (5). Kidneys are sensitive to oxygen deprivation, and renal insufficiency may occur within 24 h of BA, which if prolonged may even lead to irreversible cortical necrosis (6). Changes, such as elevation of serum creatinine (SCr) levels in early kidney function have been associated with short- and long-term consequences, including fluid overload, increased length of hospitalization, and death (7–9). Most studies focused on early renal function status of term infants, and the earliest observation time was 3 days after birth (6, 10, 11). However, in vulnerable preterm neonates, the first 24 h after birth is a critical period, and detailed renal function is beneficial for reasonable management and timely interventions, such as antibiotic dosage choice and parenteral nutrition. Studies on the early renal function status of preterm neonates of different GAs within 24 h after the development of BA are rare. Therefore, this study aimed to clarify the early profiles of the conventional indicators of renal and micturition functions in preterm neonates with BA within the first 24 h after birth by comparing the levels of SCr, blood urea nitrogen (BUN), estimated creatinine clearance (eCCI), and urinary output (UOP) between preterm neonates of different GAs with and without BA, with the goal of providing advisable information for tailor clinical management. In addition, candidate therapies based on their GAs will bring these vulnerable patient populations closer to receiving precision medicine, including fluid and electrolyte management, drug choice, and for those drugs, dose selection.

## Materials and Methods

### Study Design and Population

After obtaining institutional ethical clearance (2019-KY-023-01), this retrospective, matched case-control study was conducted in a neonatal intensive care unit (NICU) of our institution between March 2014 and March 2018.

The preterm neonates diagnosed with asphyxia by an experienced neonatologist team were included as preterm neonates with observed BA. Premature neonates (no history of fetal distress and Apgar score < 7 at 5 min) were continuously included as controls until the patients in each group were evenly distributed. The exclusion criteria were as follows: (a) congenital malformation in the newborn; (b) inherited metabolic diseases in the neonate; (c) congenital renal dysplasia detected on prenatal screening; (d) family history of kidney disease; (e) maternal use of opioids (fentanyl, remifentanil, or sufentanil), captopril, indomethacin, or other drugs that affect renal function during pregnancy; (f) maternal renal dysfunction during the perinatal period; (g) severe maternal anemia during pregnancy; (h) inadequate liquid intake within 24 h after birth; and (i) use of mannitol, ibuprofen, or diuretics within 24 h after birth.

Asphyxia was diagnosed based on a delivery history of fetal distress and/or 5 min Apgar score of <7, which was defined by the World Health Organization as failure to initiate and sustain normal breathing at birth (12–14). Fetal distress was recorded in the presence of bradycardia (fetal heart rate [FHR] <100 beats/min for >3 min) or persistent severe variable or late decelerations (persistent recording of a reduction in FHR by >15 beats/min from baseline for more than 15 s of recording with associated decreased beat-to-beat variability or reduction of FHR to <100 beats/min for >1 min) during fetal heart rate monitoring (15). GA was determined based on the date of the last menstrual period reported by the mother and on the modified Ballard maturational scoring system. Maternal complications were recorded, including eclampsia; hemolysis, elevated liver enzymes, and low platelets (HELLP) syndrome, diabetes mellitus; hypothyroidism; placental abruption; intrapartum hemorrhage; and neonatal complications including shock, sepsis, respiratory distress syndrome, and multiple organ dysfunction, were recorded.

All observed premature neonates with BA and controls were admitted to the NICU within 30 min after birth, managed by the same experienced neonatology team, given timely treatment, and guaranteed standard input, but they did not received diuretics during the observation.

### Data Collection

Emergency care and resuscitation procedures took precedence over any other procedure. All neonates were uniformly managed as per standard NICU guidelines. All patient details were treated with the strictest confidence. The neonate's gender, GA, birth length, weight, and head circumference, 1 and 5 min Apgar scores, fluid intake, and UOP within 24 h after birth were recorded. Serum samples were isolated from the peripheral vein blood for routine admission examination within the first hours after birth. Then, serum concentrations of creatinine and BUN were determined by enzymatic assay using an automatic biochemical analyzer (Beckman Coulter, Miami, FL, USA). The eCCI rate was calculated using the Schwartz formula: eCCI (mL/min/1.73 m^2^) = [0.34 × length (cm)]/SCr (mg/dL) (1 μmol/L = 0.0113 mg/dL) (16). UOP was systematically measured based on diaper weight every 3 h. The volumes of UOP and fluid intake within 24 h were obtained from the nursing records. We also analyzed the incidences of SCr > 133 μmol/L (6, 17), eCCI < 16 mL/min/1.73 m^2^ (11), and oliguria (UOP < 1.0 mL/kg/h for 24 h) (18). The material's information, including primiparity, delivery mode, gestational complication, and perinatal SCr level was recorded.

### Sample Size Calculation

The sample size was calculated and showed that examining 128 neonates would have a power of 80% in detecting a significant difference in the SCr level using a two-sided F-test with a confidence level of 95% (type 1 error, 0.05).

### Statistical Analysis

Quantitative variables are expressed as median (interquartile range), whereas categorical variables are expressed as absolute frequency (percentage). The neonate characteristics were compared between the preterm BA and preterm control groups. Continuous variables with normally distributed values (i.e., eCCI) were compared using the independent Student' *t*-test, whereas those with non-normally distributed values (i.e., SCr, BUN, and UOP) were compared using the Mann-Whitney U-test. The chi-square or Fischer's exact test (as appropriate) was used to compare dichotomous variables between the groups. Univariate logistic regression analysis was performed to evaluate the potential associations of demographic and clinical variables with deteriorating renal function risk. A *p* < 0.05 was considered to indicate statistical significance. Statistical analysis was conducted using SPSS version 23.0 (IBM Corp., Armonk, NY, USA).

## Results

### Study Population

Between March 2014 and March 2018, 2,977 hospitalized preterm neonates were treated in the NICU. Seventy-one preterm neonates had a history of fetal distress and an Apgar score <7 at 5 min. After screening by the exclusion criteria, 64 preterm neonates with BA (BA group), including 32 very preterm neonates (GA: 28–31 weeks) and 32 mid-late preterm neonates (GA: 32–36 weeks), were enrolled in the observation group. Sixty-four GA-matched preterm neonates without BA were included as controls until patients in each group were evenly distributed. All 128 patients had NICU admission charts available for review, and their mothers had normal SCr levels.

Demographic and clinical characteristics of the neonates and their mothers are summarized in Table 1. No significant differences in gender, gestational age, birth weight, birth length, 24 h fluid intake, and perinatal complications were observed between the BA and control groups. No significant differences in primiparity, gestational complications, and SCr level of mothers were found between the matched groups. No abnormal SCr levels was observed in all 128 mothers throughout their pregnancy. Gestational complications, including hypertension/preeclampsia/HELLP syndrome, diabetes, abnormal thyroidal function, placental abruption, and postpartum hemorrhage occurred in 41/64 (64.06%) mothers of preterm neonates with BA and 46/64 (71.88%) mothers of control neonates (*p* = 0.260). The vaginal delivery rate was higher in the BA group than in the control group (*p* = 0.040).

**Table 1:** Demographic and clinical characteristics of preterm neonates and their mothers.

**Items**			**Preterm neonates with BA (*****n*** **=** **64)**		**Control (*****n*** **=64)**		***P*-value**
			**28–31 week (*n* = 32)**	**32–36 week (*N* = 32)**	**28–31 week (*n* = 32)**	**32–36 week (*n* = 32)**	
Mother baseline	Primiparity	Yes	37		39		*p =* 0.72
			18	19	18	21	
	Delivery mode	Nature labor	10^*^		21		*p =* 0.04
			8	2	12	9	
	Gestational complication	Yes	41		46		*p =* 0.26
			22	19	22	24	
	Serum creatinine (μmol /L)		49.50		47.10		*p =* 0.416
			49.50	49.75	47.25	47.1	
Neonatal baseline	Gender	Male	34		37		*p =* 0.86
			18	16	19	18	
	GA (weeks)		32.00 ± 2.79		32.00 ± 2.52		*p =* 1.00
			30.00 ± 1.50	34.00 ± 1.70	29.87 ± 1.16	34.13 ± 1.52	
	Birth weight (g)		1723.00 ± 673.74		1703.90 ± 528.24		*p =* 0.86
			1232.40 ± 292.58	2207.00 ± 591.80	1335.31 ± 275.92	2072.00 ± 458.78	
	Birth length (cm)		41.42 ± 4.58		41.75 ± 4.14		*p =* 0.67
			38.31 ± 2.87	44.53 ± 3.79	38.875 ± 2.88	44.63 ± 3.09	
	24 h fluid intake (ml)		106.19 ± 37.55		100.00 ± 30.00		*p =* 0.31
			118.93 ± 43.65	93.45 ± 25.08	107.28 ± 29.18	92.71 ± 31.21	
	Perinatal complication	Yes	43		33		*p =* 0.07
			26	17	28	5	

*BA, birth asphyxia; GA, gestational age*.

* *Statistically significant difference (p < 0.05) between gestational age-matched preterm neonates with asphyxia and control preterm neonates*.

### Differences in Renal Function Between Preterm Neonates With and Without Asphyxia Within the First Day After Birth

Preterm neonates with BA had a significantly higher SCr levels (85.00 versus [vs.] 69.70 μmol/L; z = 3.61, *p* < 0.001), but a significantly lower eCCI rate (14.47 vs. 18.00 mL/min/1.73 m^2^; *t* = 3.51, *p* < 0.001), especially in the mid-late preterm neonates (SCr: 84.05 vs. 64.20 μmol/L; z = 4.41, *p* < 0.001; eCCI: 15.02 vs. 21.30 mL/min/1.73 m^2^; z = 3.57, *p* < 0.001) than GA-matched controls. However, no significant difference was found in the SCr levels and eCCI rate between the very preterm neonates with and without BA. Although the very preterm neonates with BA had a significantly higher UOP than the GA-matched controls (2.01 vs. 1.66 mL/kg/h; z = 2.01, *p* = 0.045), there was no significant difference in the UOP between the mid-late preterm neonates with and without BA and between all preterm neonates with BA and without BA. Additionally, BA did not associated with the BUN level in all preterm neonates, including the mid-late and the very preterm neonates (Table 2).

**Table 2:** Comparison of renal function of preterm neonates with or without asphyxia within 24 h after birth.

	**Very preterm neonates (*****n*** **=** **64)**		**Mid-late preterm neonates (*****n*** **=** **64)**		**All preterm neonates (*****n*** **=** **128)**	
	**Control (*n =* 32)**	**BA (*n =* 32)**	**Control (*n =* 32)**	**BA(*n =* 32)**	**Control (*n =* 64)**	**BA (*n =* 64)**
SCr (μmol/L)	73.60 (64.10–93.28)	85.00 (67.38–106.60)	64.20 (58.15–73.80)	84.05 (72.00–114.25)^*^	69.70 (60.80–83.30)	85.00 (70–109.34)^*^
BUN (mmol/L)	5.72 (3.86–7.49)	5.60 (3.93–6.90)	4.82 (3.66–5.80)	4.88 (4.03–7.66)	5.26 (3.75–6.84)	5.00 (4.00–6.50)
eCCI (mL/min/1.73 m^2^)	14.88 (12.42–18.65)	14.16 (10.08–16.90)	21.30 (17.61–24.16)	15.02 (11.76–19.23)^*^	18.00 (13.92–22.95)	14.47 (11.27–22.17)^*^
UOP (mL/kg/h)	1.66 (1.17–2.40)	2.01 (1.56–3.04) ^*^	1.45 (1.11–2.19)	1.18 (0.83–2.17)	1.58 (1.18–2.31)	1.71 (1.07–2.44)

*BA, birth asphyxia; BUN, blood urea nitrogen; eCCI, estimated creatinine clearance; SCr, serum creatinine; UOP, urinary output*.

* *Statistically significant difference (p < 0.05) between gestational age-matched preterm neonates with asphyxia and control preterm neonates*.

### Incidences of Changed Parameters Related to Renal Function in Preterm Neonates With Asphyxia Within 24 h After Birth

Within 24 h after birth, in all 64 preterm neonates with BA, the incidences of SCr > 133 μmol/L, CCI < 16 L/min/1.73 m^2^ and UOP < 1.0 ml/kg/h were 10.94% (7/64), 62.50% (40/64), and 20.31% (13/64), respectively. There was no significant difference in the incidences of SCr > 133 μmol/L (10.94% [7/64] vs. 1.56% [1/64] and oliguria (UOP < 1.0 mL/kg/h; 20.31% [13/64] vs. 15.63% [10/64]) between the 64 preterm controls and 64 preterm neonates with BA. However, the incidence of eCCI < 16 mL/min/1.73 m^2^ was significantly higher in the 64 preterm neonates with BA than in the preterm controls (62.50 % vs. 37.50%; χ^2^ = 8.00, *p* = 0.005), especially in the 32 mid-late preterm neonates (56.25% [18/32] vs. 15.63% [5/32]; χ^2^ = 11.47, *p* = 0.001). The incidence of eCCI < 16 mL/min/1.73 m^2^ was similar in the very preterm neonates with and without BA (68.75% [22/32] vs. 59.38% [19/32], respectively; χ^2^ = 0.61, *p* = 0.430 (Table 3).

**Table 3:** Incidences of changed parameters related to renal function in asphyxiated preterm neonates within 24 h after birth.

**Abnormal renal function indicators**	**Very preterm neonates**		**Mid-late preterm neonates**		**Preterm neonates**	
	**Control (*n =* 32)**	**BA (*n =* 32)**	**Control (*n =* 32)**	**BA (*n =* 32)**	**Control (*n =* 64)**	**BA (*n =* 64)**
SCr ≥ 133 μmol/L	1 (3.13%)	2 (6.25%)	0 (0%)	5 (15.63%)	1 (1.56%)	7 (10.94%)
eCCI ≤ 16 mL/min/1.73 m^2^	19 (59.38%)	22 (68.75%)	5 (15.63%)	18 (56.25%)^*^	24 (37.50%)	40 (62.50%)^*^
UOP < 1.0 mL/kg/h	3 (9.38%)	1 (3.13%)	7 (21.88%)	12 (37.50%)	10 (15.63%)	13 (20.31%)

*BA, birth asphyxia; eCCI, estimated creatinine clearance; SCr, serum creatinine; UOP, urinary output*.

* *Statistically significant difference (p < 0.05) between gestational age-matched preterm neonates with asphyxia and control preterm neonates*.

### Predisposing Factors of Deteriorating Renal Function in Preterm Neonates Within 24 h After Birth

In the univariate logistic regression analysis, asphyxia (*p* = 0.016; odds ratio [OR]= 2.83; 95% confidence interval [CI]: 1.21–6.61), perinatal complications of neonates (*p* = 0.003; OR = 3.08; 95% CI: 1.48–6.43), Preterm birth with very small GA (GA < 32 weeks) (*p* = 0.001; OR = 3.41; 95% CI: 1.65–7.06), and natural birth (*p* = 0.002; OR = 4.91; 95% CI: 0.17–9.84) were significantly associated with an increased risk of eCCI < 16 mL/min/1.73 m^2^ in all preterm neonates within the first hours after birth. Preterm birth with very small GA (<32 weeks) (*p* = 0.002; OR = 0.16; 95% CI: 0.05–0.50) and gestational complications (*p* = 0.0260; OR = 0.35; 95% CI: 0.14–0.88) were inversely associated with UOP < 1.0 ml/kg/h in preterm neonates within 24 h after birth. Primiparity and gender were not significantly associated with the change of renal function in preterm neonates within 24 h after birth (Table 4).

**Table 4:** Maternal and neonatal risk factors for early deteriorating renal function in preterm neonates.

**Parameter**		***n***	**SCr ≥ 133 μmol/L**	**Univariate logistic regression**		**UOP < 1.0 ml/kg/h**	**Univariate logistic regression**		**eCCI < 16 mL/min/1.73 m^**2**^**	**Univariate logistic regression**	
			**Yes *N =* 8**	**OR (95% CI)**	***P*-value**	**Yes *N =* 23**	**OR (95% CI)**	***P*-value**	**Yes *N =* 29**	**OR (95% CI)**	***P*-value**
Primiparity	Yes	76	4	0.70 (0.52–2.80)	0.579	17	2.21 (0.81–6.05)	0.120	38	0.68 (0.52–1.53)	0.683
Delivery mode	Nature	31	1	0.43 (0.05–3.63)	0.437	5	0.84 (0.29–2.50)	0.759	7	4.91 (0.17–9.84)	0.002^*^
Gestational complication	Yes	87	7	3.5 (0.42–29.43)	0.246	11	0.35 (0.14–0.88)	0.026^*^	45	1.62 (0.78–3.37)	0.196
Gender	Male	71	6	2.54 (0.49–13.09)	0.260	12	0.85 (0.34–2.10)	0.726	34	1.21 (0.60–2.43)	0.594
GA	<32 week	64	3	0.58 (0.13–2.54)	0.470	4	0.16 (0.05–0.50)	0.002^*^	53	3.41 (1.65–7.06)	0.001^*^
Birth asphyxia	Yes	64	8	7.74 (0.92–64.83)	0.059	13	1.38 (0.56–3.42)	0.491	40	2.83 (1.21–6.61)	0.016^*^
Perinatal complication	Yes	76	8	5.17 (0.62–43.38)	0.130	12	0.69 (0.28–1.73)	0.439	46	3.08 (1.48–6.43)	0.003^*^

*CI, confidence interval; eCCI, estimated creatinine clearance; OR, odds ratio; SCr, serum creatinine; UOP, urinary output*.

* *The p < 0.05*.

## Discussion

Our research showed that the renal function of preterm neonates was altered after BA in the first hours after birth, and different renal function profiles were observed in neonates of different GAs after BA in the first day of life. In mid-late preterm neonates after BA, a higher initial SCr value and lower initial eCCI were observed. In the very preterm neonates after BA, higher UOP was seen within the first day after birth. The incidences of SCr > 133 μmol/L, eCCI < 16 mL/min/1.73 m^2^ within the first hours after birth, and UOP < 1.0 ml/kg/h on the first day after birth were 10.94, 62.50, and 20.31%, respectively, in preterm neonates after BA. The influencing factors of early renal function in preterm neonates included BA, GA, maternal complication, and postnatal complications of neonates. Preterm birth with very small GA (<32 weeks) was inversely associated with UOP < 1.0 ml/kg/h. BA was associated with an increased incidence of eCCI < 16 mL/min/1.73 m^2^, at an OR of 2.83. Therapies based on the different renal function statuses may bring these vulnerable patient populations of different GAs closer to receiving precision medicine after BA.

In preterm neonates hospitalized in the NICU, the incidence of acute kidney injury (AKI), functional renal failure, and intrinsic renal failure were reported to be 12, 48.14, and 51.85% respectively (19). despite the great advances in neonatal resuscitation, many preterm infants still suffer from asphyxia, which is an important risk factor for impaired renal function in preterm neonates (20). Severe asphyxia leads to diffuse tubular dysfunction, which results in impaired water and sodium reabsorption and decreased GFR (21). The damage caused by impaired renal function-related complications is significantly more severe in most immature neonates with impaired glomerulogenesis (2–4, 22). Therefore, it is critical to early identify the deterioration in renal function associated with asphyxia in these vulnerable premature neonates of different GAs within the first day of life, which would bring these vulnerable patient populations closer to receiving timely precision treatment and achieving a better outcome. These treatments included fluid and electrolyte management, drug choice (avoid nephrotoxic drugs and potential nephroprotective interventions), and for those drugs, dose selection (16).

Evaluating kidney injury in preterm neonates is challenging, but it is relevant for postnatal care and crucial for the future of a patient. Although the definitions of kidney impairment and AKI staging in adults and children have become widely adopted, no single-accepted definition exists for AKI in neonates, and no single accepted definition exists for AKI in neonates and no definition has been proposed for preterm neonates (4). Additionally, the measurement of preterm neonatal renal function is challenging (16, 20). GFR represents the most recognized measure of kidney function. Although the clearance rate of inulin is the gold standard of GFR, it was difficult to measure it because intravenous pre-infusion, followed by continuous infusion of insulin, was needed. The GFR of neonates is generally based on the eCCI (23). Accurate, easy-to-use markers to estimate the actual GFR of neonates are lacking. In clinical practice, the commonly used endogenous glomerular filtration markers are SCr and BUN, especially in developing countries such as China. However, SCr and BUN are affected by extrarenal factors (e.g., muscle mass, intake of nitrogen, and protein) and associated with age, sex, and body weight (20). The reference intervals for SCr, BUN, and eCCI in preterm neonates of each gestational age have been rarely reported until now.

In the context of the above challenges, studies on the early renal function profiles of asphyxiated preterm neonates with different GAs within the first day after birth have been rare. In this study, widely used renal function indicators, namely, SCr, BUN, eCCI, and UOP, were studied and compared between preterm neonates with BA and their GA-matched controls. Considering GAs related to renal function development, the renal function profiles of the very and mid-late preterm neonates with BA were separately observed. Our results suggest that the renal function of preterm neonates with BA change just a few hours after birth and that their early renal function profiles within the first day after birth were related to GAs. Changes in SCr levels and eCCI value in mid-late preterm and UOP in very preterm neonates should be carefully monitored. Within the first few hours after birth, higher SCr levels but lower eCCI values have already been observed in mid-late preterm neonates with BA. The very preterm neonates with BA only showed higher UOP within the first 24 h after birth. Both the very and mid-late preterm neonates showed no asphyxia-related change in the initial BUN level in this study. Pan et al. also reported on the renal function status of asphyxiated preterm neonates (GA ≤ 34 weeks) at 24 h after birth, and similar SCr, BUN, and UOP values but a reduced eCCI value was observed (20). The differences in changes of renal function parameters between the above two studies may be related to the different time points of serum collection and the composition of GAs. Precision medicine is urgently needed in early renal function evaluation for the vulnerable preterm neonates of different stages of development. SCr values reflected a pattern with an initial increase and subsequent decrease during postnatal life, as observed in a cohort of 1,140 neonates (GAs 23–42 weeks) in the first 42 days of postnatal life (16, 24). In preterm neonates with gestational age ≥32 weeks, the SCr level may increase for the first several days following birth and the degree and duration of the increase is proportional to the degree of prematurity. This increase in creatinine is thought to be secondary to tubular resorption of creatinine by the immature kidney, compounded by the total body fluid loss and intravascular volume contraction typically encountered in preterm neonates (25, 26). Absolute SCr ≥133 μmol/L on the second or third day of life were frequently proposed to indicate AKI in most neonatal AKI studies (19, 27). All mothers in this study had normal SCr levels during pregnancy, labor, and delivery. The renal function of preterm neonates whose SCr levels have already been ≥133 μmol/L within the first day after birth should be paid more attention to. Therefore, we evaluated the incidence of SCr level ≥ 133 μmol/L according to routine examination results on admission and found that 10.94% of preterm neonates with BA had already shown an SCr level ≥ 133 μmol/L within the first hours after birth. However, the incidence of SCr ≥ 133 μmol/L in these neonates was not significantly different from that in their GA-matched controls. More sensitive cut-off values of creatinine in preterm neonates of different GAs still need to be explored. Thus far, no unifying accepted cut-off values of eCCI or GFR exist for preterm neonates of different GAs. Kastl (24) reported that GFR measured by renal inulin clearance may be as low as 10–20 mL/min/1.73 m^2^ at birth, and a rapid increase is then seen within the first 14 days of life and GFR is typically between 35 and 45 mL/min/1.73 m^2^. VIEUX et al. (28) reported that median GFR reference values in infants aged 27 to 31 weeks' gestation ranged from 7.9 to 30.3 mL/min per 1.73 m^2^ on day 7. In the study conducted by Nkidiaka et al. (11), AKI was diagnosed within 3 days of life when the eCCI was ≤ 16 mL/min/1.73 m^2^ and occurred in 42.85% of the 70 full-term neonates with perinatal asphyxia. Considering that very few studies have explored the cut-off values of eCCI or GFR in preterm neonates within the first day, a tentative comparison of the incidence of eCCI ≤ 16 mL/min/1.73 m^2^ was conducted between the preterm neonates with and without BA in our present study. We found that the eCCI ≤ 16 mL/min/1.73 m^2^ occurred in 62.50% of the 64 preterm neonates with BA within the first hours after birth, when compared to GA-matched preterm neonates without BA, and eCCI ≤ 16 mL/min/1.73 m^2^ was significantly seen more often in the preterm neonates with BA, especially the mid-late preterm neonates with BA. Compared to the occurrence of SCr ≥ 133 μmol/L, the occurrence of eCCI <16 mL/min/1.73 m^2^ may have more potential as an index for the early evaluation of renal function of preterm neonates after the development of BA, considering that the eCCI contains much information related to GAs and SCr reabsorption in the proximal tubules varies according to the prematurity level (22). Further studies of more preterm neonates should be conducted to detect more precise interval range values of eCCI. Oliguria is common after perinatal asphyxia, which is a manifestation of renal dysfunction. This is due to the reduction of cardiac output or AKI secondary to renal tubular necrosis (21).

The Acute Kidney Injury Network (AKIN) criteria; the modified pediatric risk, injury, failure, loss, and end-stage (RIFLE) kidney disease criteria, and the Kidney Diseases: Improving Global Outcomes (KDIGO) criteria were used in numerous researches for the diagnosis of neonatal AKI based on the increase in SCr levels and/or decrease in UOP, and UOP < 1.0 ml/kg/h was the most often used cut-off value for oliguria (17, 29). Almost all neonates urinate within 24 h after birth (19). However, the prognosis is poor, and the mortality rate is high if oliguria develops (4). Therefore, in our present study, a tentative comparison of the incidence of UOP < 1.0 ml/kg/h was conducted between the preterm neonates with and without BA within the first 24 h after birth. The incidences of oliguria were similar in the two groups. The very preterm neonates with oliguria should be more carefully monitored within the first 24 h after the development of BA.

BA has been often reported as one of the most common early and late causes of renal function decline in neonates (30). In this study, we also found that asphyxia was significantly associated with an increased risk of impaired renal function in preterm neonates within the first day after birth. BA was associated with a significantly higher incidence of eCCI < 16 mL/min/1.73 m^2^ in the preterm neonates. The potential risk factors of a higher incidence of eCCI < 16 mL/min/1.73 m^2^, including very small GA (<32 weeks), perinatal complications of neonates, and natural birth, were also observed in this study. The precise cut-off value of eCCI for neonates of different GAs needs to be further studied with a large sample in the future. Additionally, we found that preterm birth with very small GA (<32 weeks) and mothers' gestational complications were negatively associated with UOP <1.0 ml/kg/h in preterm neonates within 24 h after birth. Oliguria was not likely to occur in very preterm neonates, which may be related to the high proportion of water content in their body and the immature function of renal tubular concentration and dilution (27). Our results suggest that the influencing factors of early renal function in preterm neonates include BA, GA, maternal complication, and postnatal complications of neonates. Preterm neonates' renal function may be protected by active treatment of the basic maternal diseases during pregnancy, timely and effective asphyxia neonatorum resuscitation, and then reasonable treatment of neonatal complications.

There are several strengths and limitations of this study. Early renal function evaluation in preterm neonates of different GAs after BA within the first 24 h of life were beneficial to the vulnerable population in decreasing the risk of developing AKI within 2–3 days after birth, and better clinical outcomes were achieved after the preterm neonates received reasonable fluid and electrolyte management, drug and dose choice, and timely interventions by a neonatologist. Several measures have been taken to ensure the objectivity of this study, as all neonates were given guaranteed standard input and timely treatment; mothers' SCr levels were all normal during pregnancy, labor, and delivery; and serum was all isolated from peripheral venous blood. This study was a cross-sectional survey on the renal function profiles of preterm neonates after BA. It was difficult to obtain multiple blood samples for continuous evaluation of renal function because premature neonates have a small blood volume and very preterm neonates, especially, have a low body weight. Further research should extend the observation period to > 48 h after birth to fully clarify the early renal function profiles according to the GAs of neonates. Additionally, our sample size in this study was limited since the number of premature neonates with BA has decreased substantially owing to improvements in perinatal screening and management. Large multicenter studies are needed in the future. Finally, studies using novel and more sensitive blood or urinary markers must be conducted in the future to detect the decline in neonatal renal function as early as possible. Recently, biomarkers of renal GFR or tubular damage as predictors of early renal damage prior to SCr elevation have been explored in term and preterm neonates (16). Compared with the classic endogenous glomerular filtration markers (e.g., SCr and BUN), neutrophil gelatinase-associated lipocalin (NGAL) has emerged as a promising indicator of kidney injury. NGAL was found 34 h earlier than SCr to detect AKI (20). Protein cystatin C is not filtered by the placenta, and elevated cystatin C levels directly reflect fetal and neonate GFRs. Urinary cystatin C shows approximately 90% sensitivity and >80% specificity in predicting AKI in preterm neonates, meaning that these measures have excellent reliability for positively identifying disease and ruling out the possibility for not having the disease (31). Moreover, cystatin C had good distinguishability between asphyxiated and non-asphyxiated preterm neonates, irrespective of GA (<28, 28–32, or ≥32 week subgroups), and further discriminated between mild, moderate, and severe asphyxia (16).

Kidneys are sensitive to oxygen deprivation, and renal insufficiency may occur within 24 h of BA. Accurate evaluation of renal function status remains difficult in the newborn period. Thus far, in clinical practice, commonly used endogenous glomerular filtration markers have still been SCr, BUN, and UOP. Our results based on these classic biomarkers are helpful for understanding the asphyxia related to the early renal function profile of preterm neonates with different developmental maturity statuses, especially in developing countries. When the early renal function assessment is conducted in preterm neonates with BA within the first 24 h after birth, the changes in SCr levels and eCCI should be monitored more intensively in mid-late preterm neonates with BA, whereas oliguria should be carefully monitored in very preterm neonates. Early identification of deterioration of renal function associated with asphyxia and tailored clinical care and pharmacotherapy of the vulnerable preterm neonates of different GAs are beneficial to improve preterm neonate outcomes.

## Data Availability Statement

The raw data supporting the conclusions of this article will be made available by the authors, without undue reservation.

## Ethics Statement

This study was reviewed and approved by the Ethical Review Commitment (2019-KY-023-01) of Beijing Obstetrics and Gynecology Hospital, Capital Medical University, Beijing, China. The clinical and research activities being reported are consistent with the principles of the Declaration of Helsinki. Written informed consent from the participants' legal guardian/next of kin was not required to participate in this study in accordance with the national legislation and the institutional requirements.

## Author Contributions

YZ and H-HZ contributed to the study conception and design. Data collection was performed and the first draft of the manuscript was written by YZ. Data analysis was performed by H-HZ. All authors commented on previous versions of the manuscript, read, and approved the final manuscript.

## Conflict of Interest

The authors declare that the research was conducted in the absence of any commercial or financial relationships that could be construed as a potential conflict of interest.

## Abbreviations

AKI: acute kidney injury
BA: birth asphyxia
BUN: blood urea nitrogen
eCCI: estimated creatinine clearance
GAs: gestational ages
GFR: glomerular filtration rate
NICU: neonatal intensive care unit
SCr: serum creatinine
UOP: urinary output.
